# Personalized Oncology: Organizing Cancer Treatment Around Patient Biology

**DOI:** 10.3390/cancers18172778

**Published:** 2026-08-27

**Authors:** Julianna Lisziewicz, Oliver Wueseke, Franco Lori

**Affiliations:** 1 VERDI Solutions GmbH, 1010 Vienna, Austria; 2 ImmuLogics GmbH, 48155 Muenster, Germany; oliver.wueseke@immulogics.com

**Keywords:** personalized oncology, precision oncology, patient biology, tumor biology, cancer vaccines, immunotherapy, artificial intelligence, personalized treatment, rare cancers

## Abstract

Clinical guidelines remain the foundation of evidence-based oncology, translating population-derived evidence into treatment recommendations for defined patient groups. However, patients with rare cancers, complex tumor biology, or treatment-refractory disease may reach a point at which applicable evidence is limited, or guideline-recommended treatment options have been exhausted. We propose Personalized Oncology as a complementary framework for treatment selection in these situations. Rather than asking only which treatments benefited similar patients, Personalized Oncology asks which available treatment is expected to provide the most favorable benefit–risk profile for the individual patient. It does so by integrating population-derived medical evidence with patient-specific biological evidence generated from the individual cancer. Importantly, exhaustion of guideline-recommended treatments does not necessarily mean exhaustion of biologically supported treatment opportunities. The supporting evidence, rationale, alternatives, limitations, and uncertainty are documented to enable transparent clinical decision-making. Neither population-derived evidence nor patient-specific biological evidence determines in advance whether an individual patient will benefit. Treatment response must therefore be systematically monitored and incorporated into subsequent decisions. Personalized Oncology standardizes this process while preserving individualized clinical judgment. Systematic capture of each patient’s biology, treatment, and outcome can create a continuous learning framework in which experience from individual patients informs future cancer care.

## Simple Summary

Cancer treatment guidelines are based on results from groups of patients and remain the foundation of cancer care. However, some patients, especially those with rare cancers or cancers that no longer respond to standard treatments, reach a point where guidelines provide no further treatment option. This does not necessarily mean that no treatment opportunity remains. The individual patient’s cancer may have biological features that suggest additional treatment options. We propose Personalized Oncology as a framework for identifying these opportunities by combining what is already known from medical research with detailed information about the individual patient’s cancer. Because no evidence can guarantee that a treatment will work for an individual, the patient’s response is carefully monitored and used to guide subsequent treatment decisions.

## 1. Introduction

Oncology has advanced by improving how evidence is generated and applied. Early treatment decisions relied on tumor anatomy and histopathology. Randomized clinical trials established evidence-based medicine as the foundation of clinical guidelines, while precision oncology extended this paradigm by matching therapies to molecular biomarkers and actionable genomic alterations, improving outcomes for many patients [1,2].

This population-based approach has improved cancer care but cannot capture the biological complexity of every individual. When patients have rare cancers, uncommon biological features, or resistance mechanisms that fall outside established evidence-based pathways, physicians need to make treatment decisions despite limited directly applicable evidence [2,3].

Personalized Oncology is a complementary framework that integrates patient-specific biological evidence with population-derived evidence to support personalized treatment decisions. Rather than relying on a single biomarker, it combines genomic, transcriptomic, immunological, pathological, and clinical information to characterize the biology of the individual patient and disease. Its aim is not to replace clinical guidelines, evidence-based medicine, or physician judgment but to strengthen treatment decisions when directly applicable population evidence is limited.

Unlike precision oncology, which asks whether a patient belongs to a previously characterized molecular subgroup, Personalized Oncology addresses a complementary question: which available treatment is expected to provide the most favorable benefit–risk profile for this individual patient? Although each patient’s biology is unique, the process used to characterize and interpret it can be standardized. Consistent workflows, systematic interpretation, transparent documentation, and structured outcome assessment support personalized treatment decisions while enabling patient observations to contribute to a continuously learning framework that informs future care [4,5].

In this Perspective, we distinguish Personalized Oncology from precision oncology by the direction in which evidence is generated and applied; relate the framework to published precedents for its individual components, including combination strategies and personalized cancer vaccines; and discuss how standardized workflows, artificial intelligence, and continuous learning may support its implementation.

## 2. Precision Oncology

Precision oncology transformed cancer treatment by linking molecular features of tumors to therapeutic selection. Contemporary precision oncology may integrate histopathology, genomic and other molecular biomarkers, tumor-agnostic indications, clinical trials, and multidisciplinary molecular tumor-board interpretation. Its central strength is the application of population-derived evidence to increasingly refine biologically defined patient groups (Table 1). In the I-PREDICT study, a higher degree of matching between a tumor’s alterations and the therapies given was associated with longer progression-free and overall survival, illustrating the potential of biology-guided treatment within precision oncology [6,7].

Tumor-agnostic treatment strategies based on NTRK fusions and mismatch-repair deficiency illustrate how molecularly defined treatment populations can transcend histological boundaries [8,9].

As molecular data grew more complex, molecular tumor boards became a standard part of interpreting it [10]. A systematic review and a meta-analysis report improved outcomes with biomarker-matched therapy recommended by these boards, while noting substantial heterogeneity between studies and a lack of randomized evidence [11,12], and professional bodies have begun to set quality standards for how they operate [13].

The strength of precision oncology is that it generates robust evidence from populations of patients who share a biological feature. Patients are grouped by histology, biomarker, or actionable alteration, and recommendations follow from trials in similar groups. This population-based logic remains the foundation of modern oncology.

## 3. Limitations of Population-Based Evidence

Population-derived evidence cannot answer every question at the bedside. Molecular profiling has revealed wide biological heterogeneity among tumors that share a diagnosis, a biomarker, or a mutation, and the average outcome of a population does not predict the outcome of one patient. Two people with apparently similar tumors can differ in disease biology, treatment sensitivity, immune response, and survival.

This gap is widest in specific settings. Patients with rare or ultra-rare cancers often lack cohort evidence to guide treatment [14]. Patients with treatment-refractory disease may still carry biologically relevant vulnerabilities after standard options are exhausted. In both, clinicians must decide without directly applicable population evidence.

Generating evidence at the level of the individual is fully consistent with evidence-based medicine. Evidence-based medicine was never limited to randomized trials; it integrates the best available external evidence with clinical expertise and the individual patient’s circumstances [15]. When population evidence is insufficient for a given patient, using the best available evidence for that patient is precisely what the framework demands. Recent proposals extend this further, adding real-world evidence, adaptive learning, and N-of-1 approaches as complementary sources of evidence [16].

Personalized strategies bear this out. N-of-1 approaches built on comprehensive molecular characterization have shown encouraging results in rare and refractory cancers [7,14]. They also illustrate that patients who share a histological diagnosis need not share the same treatment-relevant biological configuration. As characterization deepens, the individual patient-disease system may contain vulnerabilities that are not captured by predefined biomarkers or population averages. This is the starting point for Personalized Oncology.

## 4. Personalized Oncology: From Patient Biology to Patient-Specific Evidence

What distinguishes Personalized Oncology from precision oncology is not its technology but the direction in which evidence is generated. Precision oncology applies evidence established in populations—through biomarkers, trials, and guidelines—to the individual [1,2,3]. Personalized Oncology runs the other way: it generates patient-specific biological evidence that complements population evidence for an individual decision. It does not replace evidence-based medicine; it carries out its central principle—applying the best available evidence to each patient—when population evidence alone cannot answer the question a particular patient poses [15,16].

**Figure 1:**
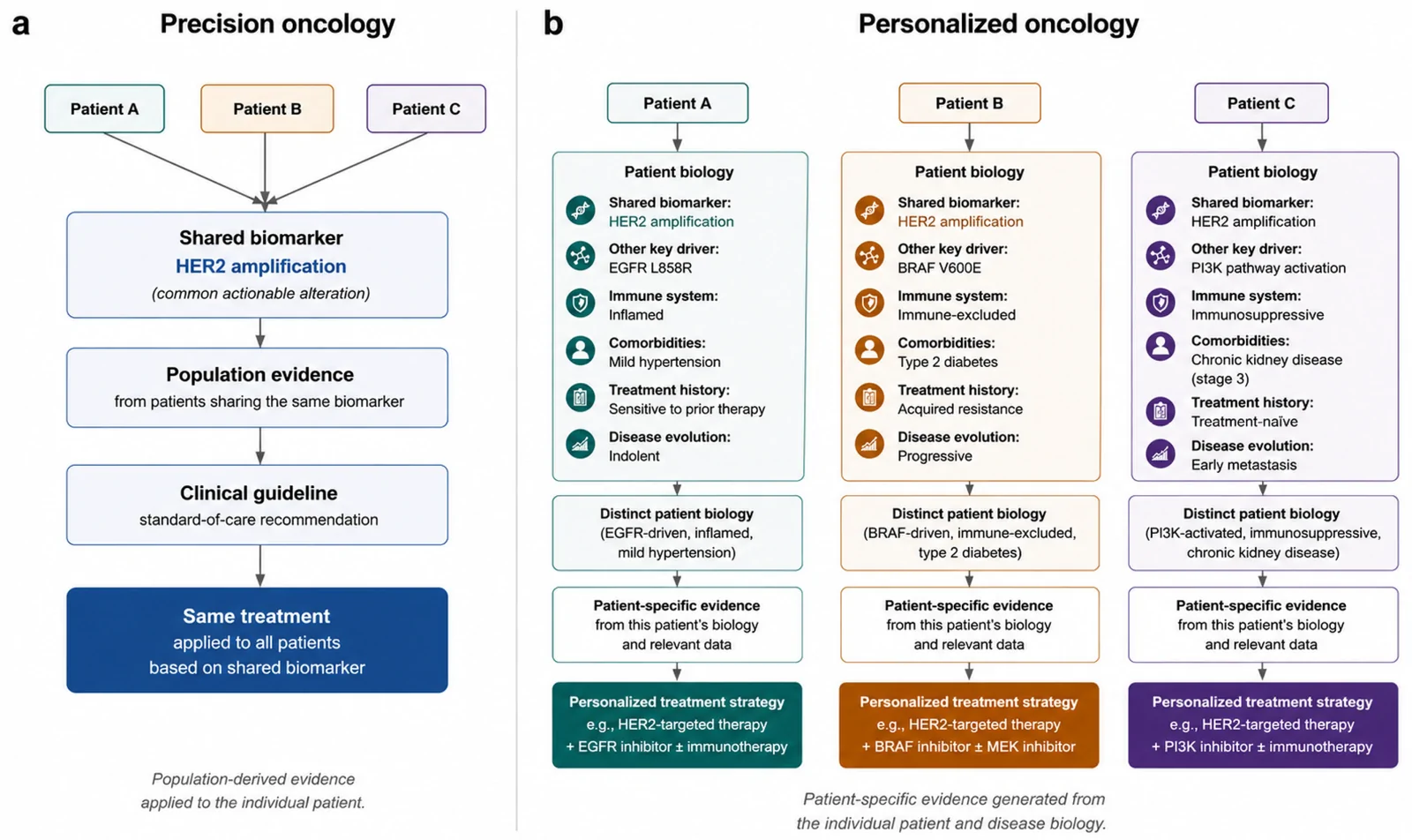
Two complementary approaches to evidence-based oncology. (**a**) Precision oncology organizes treatment around a shared biomarker and applies population-derived evidence to individual patients. (**b**) Personalized Oncology begins with the same biomarker but organizes treatment opportunities around each patient’s biology, generating patient-specific biological evidence to support personalized treatment decisions including when no matching population has previously been defined.

The distinction between precision oncology and Personalized Oncology does not depend on the technologies used (Figure 1). Both may use genomic profiling, transcriptomics, immune characterization, molecular tumor boards, and targeted treatment. The distinction lies in how the resulting information is organized for clinical decision-making. Precision oncology primarily determines whether an individual patient belongs to a previously characterized biological subgroup for which treatment evidence already exists. Personalized Oncology begins with the integrated biology of the individual patient, including biological configurations that may not correspond to any previously defined subgroup, and asks which available treatment is expected to provide the most favorable benefit–risk profile for that individual patient. Thus, the difference is not between limited and comprehensive profiling, but between organizing treatment around membership in an evidence-defined population and organizing it around the biological system of the individual patient.

In Personalized Oncology, the individual patient—not the tumor alone—is the unit of investigation. Cancer is treated as a dynamic system shaped by the tumor, the immune system, the host, prior therapies, comorbidities, disease evolution, and the current clinical context [17,18]. The question is not whether the patient fits a predefined subgroup, but what the patient’s own biology reveals about the mechanisms driving progression, resistance, immune escape, and vulnerability.

Answering this question requires comprehensive characterization of the patient–disease system. Histopathology, clinical history, genomic and transcriptomic profiling, immune biology, host biology, disease evolution, and current scientific and clinical knowledge each provide complementary information (Table 2). These sources are integrated into a structured representation of the patient’s disease biology, which we refer to as the Patient Biology Map. The Patient Biology Map is not itself evidence; rather, it is the substrate on which biological interpretation is performed.

Although these information sources are familiar to many oncologists, their growing volume, complexity, and interdependence make comprehensive and standardized interpretation difficult. Personalized Oncology provides a structured framework in which multidisciplinary expertise, computational methods, and continuously evolving scientific knowledge support consistent biological interpretation and personalized treatment assessment.

**Figure 2:**
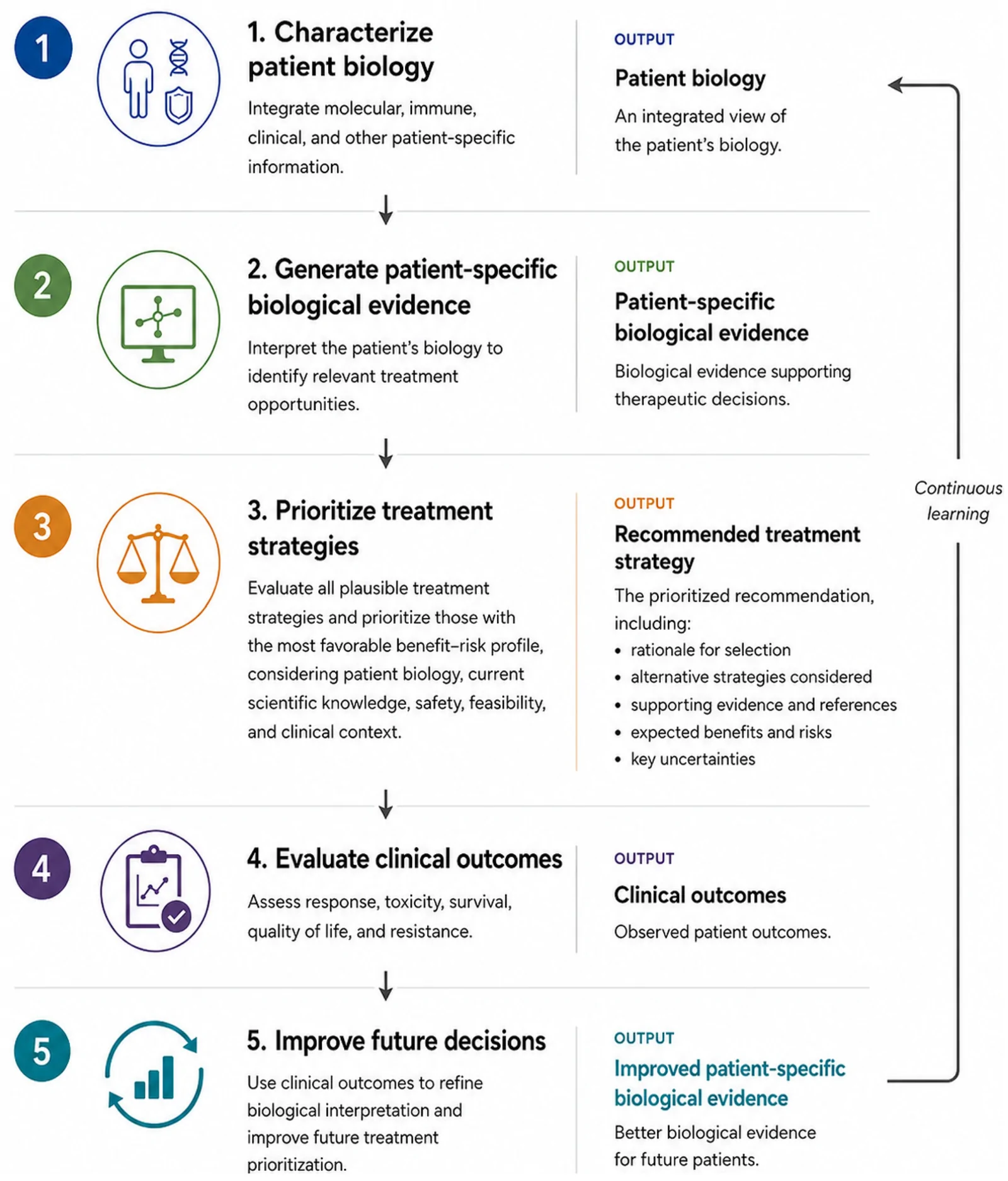
The patient-specific evidence framework for Personalized Oncology. Step 1 characterizes the patient’s biology by integrating molecular, immune, clinical, and other relevant information into a biological representation. Step 2 interprets the patient’s biology to generate patient-specific biological evidence. Step 3 evaluates biologically plausible treatment strategies and recommends the strategy with the most favorable benefit–risk profile based on patient-specific biological evidence, current scientific knowledge, safety, feasibility, and the patient’s clinical context, while documenting the alternatives considered and the scientific rationale for the recommendation. Step 4 systematically evaluates clinical outcomes, including treatment response, toxicity, survival, quality of life, and resistance. Step 5 incorporates these outcomes to refine biological interpretation and strengthen patient-specific biological evidence for future patients through continuous learning, while physician oversight is maintained throughout.

Patient-specific biological evidence is generated by interpreting the biology of an individual patient. We define it as the biological interpretation of a patient’s disease that supports treatment selection (Figure 2). Unlike evidence derived from clinical trials, which estimates the average benefit and risk of treatment across defined patient groups, patient-specific biological evidence explains the mechanisms operating in one patient and how they may influence response or resistance. A biomarker therefore becomes one input among many. The goal is not simply to identify an actionable alteration, but to integrate the patient’s biology into a coherent biological rationale for treatment selection.

What makes Personalized Oncology new is not a new technology, but the way it generates and uses evidence. It rests on a simple distinction. Population evidence tells us whether a treatment rule has been confirmed across a group of similar patients. Patient-specific biological evidence tells us whether the biology of one particular patient supports a plausible treatment opportunity. These are not the same, and neither one proves that the treatment will work—they point to an opportunity worth evaluating, with its strength and its uncertainty written down (Table 3).

Three things follow. First, patient-specific biological evidence is its own kind of evidence, separate from a validated biomarker. When a validated biomarker is missing, it does not mean there is no biological evidence in that patient—only that the relationship has not yet been confirmed in a large population. Second, every patient goes through the same process: study the biology, decide, record the outcome, and learn from it. Third, the treatments considered are not limited to standard targeted drugs but also include therapies designed for the individual patient, such as personalized vaccines.

Patient-specific biological evidence may arise from different sources, including validated biomarkers, mechanistic findings, genomic or transcriptomic signals, immune-context information, and observations generated during the patient’s own treatment course. These sources should not be forced into a single ordinal hierarchy because evidence type and validation status are different dimensions. For each treatment hypothesis, the patient-specific finding, its biological interpretation, the relevant external evidence or validation status, and the remaining uncertainty should be documented explicitly. A validated biomarker is therefore one possible source of evidence, not a prerequisite for Personalized Oncology.

Patient-specific biological evidence is necessarily evidence about the biological material and clinical state that have actually been characterized. It cannot establish that unsampled lesions or subclones share the same vulnerabilities. Spatial and temporal heterogeneity can therefore leave malignant clones that are not represented by the sampled tumor and may not respond to a treatment selected from that sample [19]. Personalized Oncology must make this limitation explicit and, when clinically feasible, use longitudinal reassessment and complementary evidence sources as the disease evolves.

The two forms of evidence are complementary. Clinical trials define the standard of care and the expected effects of treatment across patient populations. Patient-specific biological evidence explains how those expectations apply to the individual patient. Together they support treatment decisions that are grounded both in established clinical evidence and in the biology of the individual.

Both forms of evidence are predictive rather than deterministic. Clinical trials estimate the probability of benefit and harm but cannot determine whether a particular patient will respond. Patient-specific biological evidence addresses whether the biological mechanisms identified in that patient provide a rationale for expecting benefit or resistance. Personalized Oncology therefore complements guideline-based care when directly applicable clinical evidence is insufficient, integrating patient-specific biological evidence into transparent, physician-directed treatment decisions.

## 5. From Patient-Specific Evidence to Personalized Treatment

Patient-specific evidence matters only when it changes a treatment decision. Personalized Oncology therefore does not stop at describing the patient’s biology. It uses that biology to compare treatment options and identify the strategy with the most favorable benefit–risk profile.

Different treatments call for different evidence (Table 4). Surgery, radiotherapy, and chemotherapy reduce tumor burden. Antibody–drug conjugates additionally require appropriate expression of the therapeutic target and careful assessment of treatment-specific safety [20]. Targeted therapy requires evidence relevant to the pathway or molecular vulnerability being targeted, and genomic and transcriptomic profiling can provide complementary information for treatment selection [21]. Immunotherapy requires consideration of tumor–immune biology, including antigenicity and immune context [22]. Personalized cancer vaccines additionally require selection of antigens capable of generating patient-specific T-cell responses [23,24,25,26,27,28]. Treatment planning therefore rests not on one biomarker but on the lines of evidence relevant to each therapeutic decision.

Because cancer is driven by interacting biological processes and may contain coexisting sensitive and resistant clones, the preferred strategy may include more than one treatment modality. Rational combinations can be considered when patient-specific biology supports complementary therapeutic opportunities or when a single intervention is unlikely to address the relevant heterogeneity or resistance mechanisms [29]. The aim is not to maximize the number of treatments, but to address complementary mechanisms while maintaining an acceptable benefit–risk profile and explicitly accounting for toxicity, feasibility, sequencing, and uncertainty.

Patient-specific evidence does not establish one universally correct treatment. It enables relevant strategies to be compared, alternatives to be documented, and the recommendation with the strongest integrated biological and clinical support to be justified.

## 6. From Individual Patients to a Continuously Learning Clinical Framework

At first glance, Personalized Oncology appears impossible to scale. Every patient is biologically unique, so every treatment decision seems unique. The solution is not to standardize patients. It is to standardize how patient-specific biological evidence is generated.

**Figure 3:**
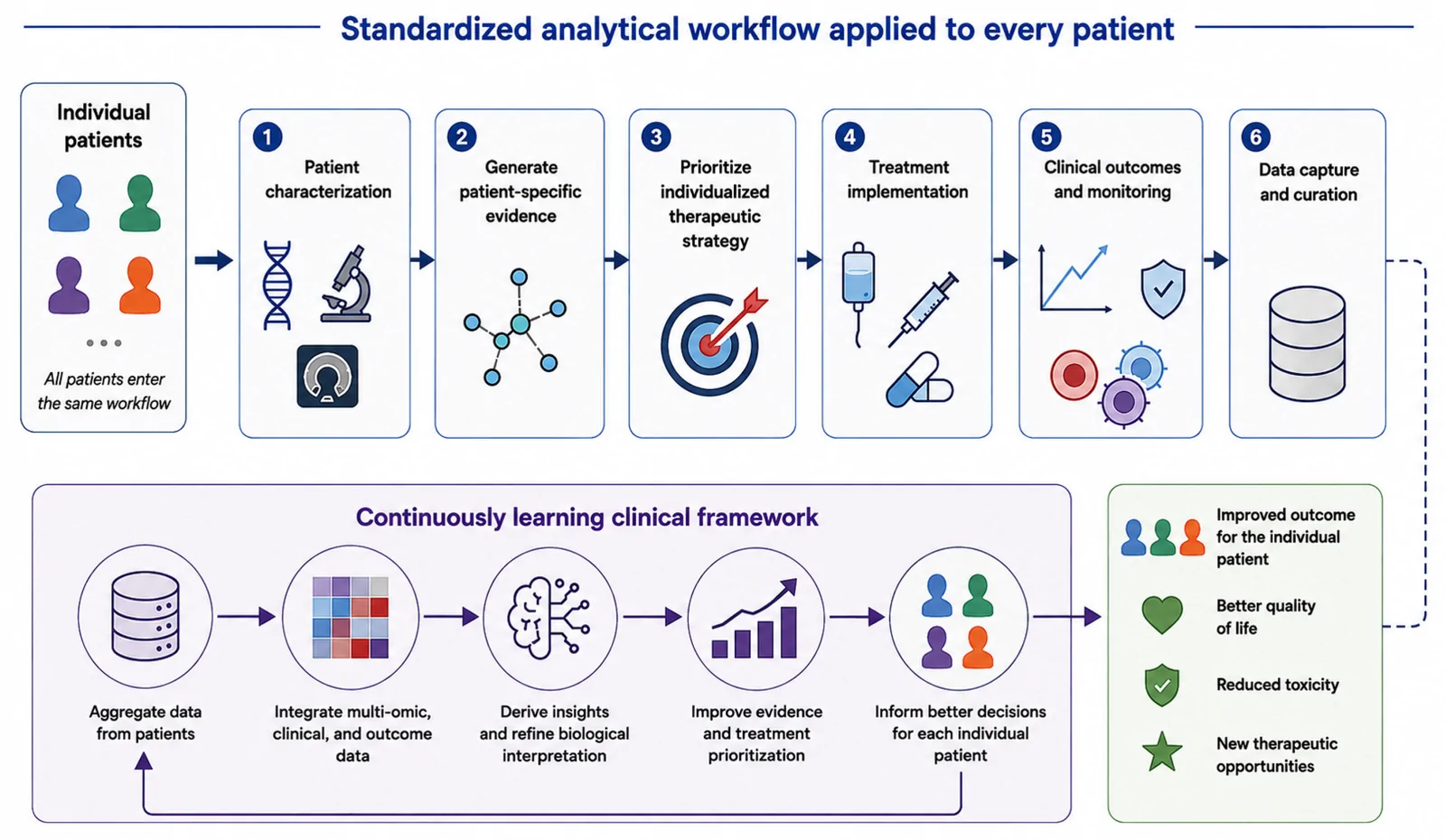
From individual patients to a continuously learning clinical framework. The workflow shown here is the clinical implementation of the evidence framework in Figure 2, with treatment implementation and structured data capture shown as separate operational steps. Each patient undergoes the same standardized workflow, beginning with comprehensive characterization of the patient–disease system. The resulting information is integrated into a structured representation of the patient’s disease biology. Biological interpretation generates patient-specific evidence, which prioritizes personalized therapeutic strategies according to the patient’s biology and clinical context. After treatment, clinical outcomes—response, survival, toxicity, immune monitoring, and quality of life—are systematically evaluated and linked back to the corresponding biology and decisions. Aggregated across many patients, these structured biology–decision–outcome records form an expanding knowledge base that continuously refines interpretation and prioritization for future patients.

Every patient enters the same clinical framework (Figure 3). Biological information is integrated into a Patient Biology Map. The map is interpreted to generate patient-specific biological evidence. That evidence supports treatment selection, and the biological prediction is evaluated against the observed clinical outcome.

This distinction changes the role of every treated patient. In conventional evidence-based medicine, patients primarily receive evidence generated in clinical trials. In Personalized Oncology, every patient also contributes new patient-specific biological evidence. As Patient Biology Maps, treatment decisions, and clinical outcomes accumulate, they progressively refine biological interpretation and improve future treatment selection.

Artificial intelligence may become an important enabling technology for Personalized Oncology because the framework requires integration of heterogeneous patient-specific biological information with rapidly expanding biomedical knowledge. Potential applications include evidence retrieval and synthesis, integration of genomic, transcriptomic, proteomic and immunological information, recognition of biological patterns across patients, standardized documentation, and longitudinal comparison of predicted with observed outcomes [30]. AI can support scale and consistency, but it is not itself a source of clinical evidence and does not replace physician judgment or clinical responsibility.

The same principle can support scalability. Reusable computational infrastructure and cloud-based genomic analysis can facilitate complex molecular workflows and collaboration [31]. Within Personalized Oncology, standardized assays, analytical pipelines, reporting systems, and, where applicable, manufacturing platforms could similarly provide reusable infrastructure that is refined as experience accumulates.

Scalability, however, requires rigorous governance. Because patient-specific biological evidence can influence treatment selection, the process by which it is generated must be transparent, reproducible, and auditable. Every recommendation should document the biological rationale, relevant external evidence, uncertainty, available alternatives, expected benefit and toxicity, and should remain subject to informed consent, toxicity monitoring, data privacy protections, and systematic outcome evaluation. Multidisciplinary review, including molecular tumor boards where available, can provide an important governance and quality-control mechanism by independently assessing the evidence, therapeutic rationale, uncertainty, safety, feasibility, and alternatives. Personalized Oncology is not defined by the molecular tumor board, however, because the evidence considered may extend beyond validated biomarkers or guideline-linked genomic alterations.

The same principle applies to personalized therapeutics. For approved medicines, manufacturing quality, pharmacology, and safety profiles remain unchanged. Personalized products, such as individualized cancer vaccines, are different because each product is unique by design. Their reproducibility therefore depends not on manufacturing identical products, but on applying the same validated and auditable platform.

Personalized Oncology should be judged by the same standard. Its reliability does not depend on identical treatment decisions for different patients. It depends on the reproducibility of a standardized process that consistently transforms individual patient biology into patient-specific biological evidence, supports transparent treatment selection, and captures outcomes for continuous learning.

Clinical utility in Personalized Oncology should be considered at complementary levels. We distinguish treatment-selection utility, clinical outcome in the individual patient, and population-level evidence of efficacy. For a patient with limited or exhausted established treatment options, Personalized Oncology may have treatment-selection utility if patient-specific biological evidence identifies an additional biologically supported and clinically implementable treatment opportunity that would not otherwise have been considered.

Such treatment-selection utility does not establish in advance that the proposed treatment will benefit that individual patient. Importantly, population-level evidence of efficacy also does not establish that an individual patient will respond; it estimates treatment effects within a studied population, within which outcomes remain heterogeneous. Population-level clinical evidence and patient-specific biological evidence therefore provide different forms of evidence for treatment selection, with different strengths and uncertainties, but neither determines the treatment outcome of an individual patient.

Consequently, regardless of whether a treatment opportunity is supported predominantly by population-level clinical evidence, patient-specific biological evidence, or their combination, the individual patient’s response must be systematically monitored. Measurable outcomes can include objective response, duration of response, progression-free survival, toxicity, patient-reported outcomes and quality of life, and survival. Prospective framework-level studies can additionally assess the proportion of patients for whom additional treatment opportunities are identified, the proportion implemented, feasibility, turnaround time, and reproducibility across reviewers or institutions.

Personalized Oncology does not reject population-based evidence when such evidence is available. Rather, it proposes that population-level evidence or a validated tumor-specific predictive biomarker should not be an exclusive prerequisite for identifying a treatment opportunity when the relevant biology of an individual patient’s cancer is not adequately represented by existing evidence. Whether the overall clinical utility of Personalized Oncology can be evaluated adequately using conventional population-based trial designs alone is therefore an important methodological question. Comparative studies with state-of-the-art precision oncology can provide evidence at the framework level, while systematic prospective generation, documentation, and longitudinal evaluation of patient-specific evidence and outcomes will be central to validation of the concept.

Several limitations temper these prospects. The framework depends on advanced molecular testing, computational infrastructure, multidisciplinary expertise, and high-quality longitudinal data that are unevenly distributed, and differences in expertise across institutions may affect reproducibility. Cost, reimbursement, access to advanced testing, data integration, data governance and privacy, and professional responsibility for off-label or individually designed treatment remain substantial implementation challenges. These issues are not unique to Personalized Oncology, but greater individualization can increase the amount and complexity of information that must be generated, interpreted, documented, and protected. A further challenge is the clinical and ethical situation in which no satisfactory established treatment remains while patient-specific biological evidence identifies a plausible therapeutic opportunity. Absence of a population-level approval or validated tumor-specific biomarker does not necessarily mean absence of relevant evidence, but biological plausibility alone is not proof of efficacy. Such decisions therefore require explicit documentation of evidence and uncertainty, informed consent, careful benefit–risk assessment, appropriate review, and systematic outcome capture.

## 7. Published Precedents for Components of Personalized Oncology

Personalized Oncology is proposed here as a future treatment concept rather than as an already validated clinical approach. Several components required for such a framework already have precedents in published research and clinical practice, but those studies were developed for different purposes and should not be retrospectively labeled as examples or validation of Personalized Oncology. Table 5 therefore distinguishes published precedents for individual components from the additional integration proposed by this Perspective.

## 8. Perspective: Evolution of Evidence-Based Medicine

Personalized Oncology is an extension of evidence-based medicine for situations in which population-derived evidence does not adequately represent the biology of an individual patient. It integrates population-derived evidence with patient-specific biological evidence generated through comprehensive characterization and interpretation of the patient–disease system. A specialized company may perform this integration through standardized computational workflows, combining the patient’s biological and clinical data with relevant scientific and clinical knowledge to generate and document patient-specific biological evidence and identify biologically supported treatment possibilities, including personalized therapeutic options. The resulting report does not constitute a treatment recommendation. The treating oncologist, supported by tumor-board, evaluates these opportunities in the patient’s clinical context and remains responsible for selecting the strategy expected to provide the most favorable benefit–risk profile for that patient.

However, every treatment decision for an individual patient is made under uncertainty. Regardless of whether it is informed by clinical experience, peer-reviewed literature, clinical-trial evidence, patient-specific biological evidence, or a combination of these sources, no treatment decision can guarantee success for that individual patient. Personalized Oncology therefore does not replace clinical guidelines or physician judgment. It strengthens treatment selection by identifying and evaluating biologically supported opportunities and by documenting the rationale, alternatives, limitations, and uncertainty.

The broader contribution of Personalized Oncology is a standardized framework linking patient biology, biological interpretation, treatment selection, and observed clinical outcomes. Standardization applies to the process, not to patients or treatment decisions. Because treatment success cannot be known in advance, systematic outcome evaluation is an essential part of the framework. The capture of biology–decision–outcome records allow each patient’s response—including benefit, lack of benefit, toxicity, and resistance—to inform subsequent treatment decisions and refine future biological interpretation. Evidence therefore no longer flows only from clinical trials to patients; it also flows from individual patients back into a continuously learning clinical framework, while safety, uncertainty, alternatives, and clinical responsibility remain explicit.

Population-derived clinical evidence transformed modern medicine and remains a foundation of evidence-based care. Personalized Oncology adds a complementary layer of evidence generation by systematically transforming the biology of individual patients into patient-specific biological evidence. Together, these forms of evidence can support more informed and transparent treatment decisions for patients today while continuously strengthening the evidence available for patients in the future.

## 9. Conclusions

Personalized Oncology extends evidence-based medicine by complementing population-derived evidence with patient-specific biological evidence when existing evidence does not adequately represent the biology of an individual patient. Through systematic integration and interpretation of patient-specific biological and clinical information, it can identify and document biologically supported treatment possibilities, including personalized vaccine options. These possibilities do not constitute treatment recommendations; treatment selection remains the responsibility of the treating oncologist, supported where appropriate by a multidisciplinary tumor board. Because no form of evidence can determine an individual patient’s outcome in advance, systematic evaluation of real-world clinical outcomes is essential. Linking patient biology, identified treatment possibilities, clinical decisions, and observed outcomes may allow experience from individual patients to improve cancer care.

## Figures and Tables

**Table 1:** Major sources of evidence supporting precision oncology.

Evidence Source	Contribution to Clinical Decision-Making
Histopathology	Defines tumor type, grade, and diagnostic classification
Predictive biomarkers	Identifies patients likely to benefit from specific therapies
Actionable genomic alterations	Enables molecularly matched treatment selection
Clinical trials	Establishes safety and efficacy in biologically defined patient populations
Treatment guidelines	Standardizes evidence-based clinical practice and reimbursement decisions

**Table 2:** Complementary evidence sources used in Personalized Oncology and their contribution to biological interpretation and personalized benefit–risk assessment. This information is available in some oncology practices; the novelty of Personalized Oncology lies in their systematic integration into patient-specific biological evidence.

Evidence Source	Contribution
Histopathology	Tumor architecture and disease context
Clinical history	Disease course, prior treatments, clinical constraints
Tumor genomics	Driver alterations and therapeutic vulnerabilities
Tumor transcriptomics	Active pathways and target expression
Immune biology	Immune status and therapeutic opportunities
Tumor evolution	Progression and resistance mechanisms
Host biology	Factors influencing response and toxicity
Scientific and clinical knowledge	Interpretation of findings and treatment relevance

**Table 3:** Complementary questions addressed by precision oncology and Personalized Oncology.

	Precision Oncology	Personalized Oncology
Central question	Which established, molecularly defined treatment strategy applies to this patient?	What treatment opportunities can be identified from this patient’s biology?
Evidence	Population-derived evidence linked to molecular characteristics	Patient-specific biological evidence integrated with relevant external evidence
Treatment space	Molecularly targeted treatment options	Individually designed therapies including personalized vaccines
Validated biomarker	Central to treatment selection	Not required as a defining condition
Rare/unique biology	Limited when population-level validation is unavailable	Can generate treatment hypotheses from the patient’s biology, with uncertainty explicitly documented
Output	Molecularly informed treatment selection	Individual therapeutic strategy

**Table 4:** Patient-specific biological evidence supporting treatment decisions.

Evidence	Treatment Decisions	What It Tells Us
Tumor burden and location	Surgery, radiotherapy, chemotherapy	Whether cytoreduction is needed, feasible, and appropriately timed.
Molecular drivers and resistance	Targeted therapies	Which pathways should be targeted, and which resistance mechanisms may limit benefit.
Therapeutic target expression	Antibody–drug conjugates, radioligand therapies, targeted biologics	Whether sufficient target is present for target-directed treatment.
Tumor–immune biology	Immunotherapies, cancer vaccines, adoptive cell therapies	Whether anti-tumor immunity can be initiated, restored, or strengthened.
Clinical context and prior treatment	Treatment selection, sequencing, and combinations	Which strategy offers the most favorable benefit–risk profile for the individual patient.

**Table 5:** Published precedents for components integrated in the Personalized Oncology framework.

Published Precedent	Proposed Role in Personalized Oncology	Ref.
**Molecular profiling and molecular tumor boards:** identify actionable alterations and support individualized treatment recommendations.	Evaluate the benefit–risk profile and feasibility of personalized treatment options.	[6,21]
**Genomic and transcriptomic profiling:** can broaden treatment matching beyond genomic alterations alone.	Integrate biological evidence relevant to the individual treatment question.	[21]
**Individually matched drug combinations:** molecular profiling can guide multidrug treatment regimens.	Consider combinations supported by patient-specific biology, while documenting toxicity, feasibility, and uncertainty.	[6]
**Personalized cancer vaccines:** can be manufactured and administered and have demonstrated clinical benefit in selected settings.	Serve as key components of a patient-specific treatment strategy aimed at durable tumor control.	[23,24,25,26,27,28]
**Tumor heterogeneity and sampling:** a single sample may not capture all treatment-relevant tumor biology.	Restrict interpretation to the sampled material and time point and reassess as the disease evolves.	[19]
**Resistance-informed combinations:** resistant clones can limit the durability of tumor control achieved with single-agent treatment.	Consider combinations targeting complementary vulnerabilities while accounting for toxicity and feasibility.	[29]

## Data Availability

No new or unpublished datasets are reported in this Perspective. All data discussed are available within the cited publications.
